# Racial and Gender Differences in Cardiorespiratory Fitness and Atrial Fibrillation

**DOI:** 10.31083/j.rcm2507261

**Published:** 2024-07-11

**Authors:** Evan Czulada, Samir A. Shah, Apostolos Tsimploulis

**Affiliations:** ^1^School of Medicine, Georgetown University, Washington, D.C. 20007, USA; ^2^School of Medicine and Health Sciences, George Washington University, Washington, D.C. 20037, USA; ^3^Department of Electrophysiology, MedStar Heart and Vascular Institute, Washington, D.C. 20010, USA

**Keywords:** atrial fibrillation, cardiorespiratory fitness, race, gender, exercise

## Abstract

The expanding field of cardiorespiratory fitness (CRF) in individuals with and without atrial fibrillation (AF) presents a complex landscape, demanding careful interpretation of the existing research. AF, characterized by significant mortality and morbidity, prompts the exploration of strategies to mitigate its impact. Increasing physical activity (PA) levels emerges as a promising avenue to address AF risk factors, such as obesity, hypertension, and diabetes mellitus, through mechanisms of reduced vasoconstriction, endothelin-1 modulation, and improved insulin sensitivity. However, caution is warranted, as recent investigations suggest a heightened incidence of AF, particularly in athletes engaged in high-intensity exercise, due to the formation of ectopic foci and changes in cardiac anatomy. Accordingly, patients should adhere to guideline-recommended amounts of low-to-moderate PA to balance benefits and minimize adverse effects. When looking closer at the current evidence, gender-specific differences have been observed and challenged conventional understanding, with women demonstrating decreased AF risk even at extreme exercise levels. This phenomenon may be rooted in divergent hemodynamic and structural responses to exercise between men and women. Existing research is predominantly observational and limited to racially homogenous populations, which underscores the need for comprehensive studies encompassing diverse, non-White ethnic groups in athlete and non-athlete populations. These individuals exhibit a disproportionately high burden of AF risk factors that could be addressed through improved CRF. Despite the limitations, randomized control trials offer promising evidence for the efficacy of CRF interventions in patients with preexisting AF, showcasing improvements in clinically significant AF outcomes and patient quality of life. The potential of CRF as a countermeasure to the consequences of AF remains an area of great promise, urging future research to delve deeper to explore its role within specific racial and gender contexts. This comprehensive understanding will contribute to the development of tailored strategies for optimizing cardiovascular health and AF prevention in all those who are affected.

## 1. Introduction

Atrial fibrillation (AF) is the most common cardiac arrhythmia, and its prevalence is increasing in the United States (US) and worldwide. In 2010, the US prevalence of AF was 5.2 million; by 2030, that number is expected to rise to 12.1 million [1]. AF is a condition with significant mortality and morbidity [2], and importantly, AF is often seen in conjunction with other known cardiovascular risk factors, such as hypertension (HTN), obesity, diabetes mellitus (DM), and dyslipidemia. As such, modification of risk factors is one of the principal pillars of preexisting AF management in the most recently published 2023 American Heart Association (AHA)/American College of Cardiology (ACC)/Heart Rhythm Society (HRS) guidelines, which also highlights risk factor reduction as an important method for primary AF prevention [3]. In fact, mitigating these determinants can be especially effective in certain populations that face higher burden and increased severity of AF due to other underlying conditions [4]. One way to address this issue is by promoting lifestyle changes, one of the most powerful tools being exercise.

Improving cardiorespiratory fitness (CRF) and physical activity (PA) are highly effective strategies in preventing AF [5]. AF is related to and often worsened by existing comorbidities, such as HTN, coronary artery disease (CAD), heart failure (HF), obesity, and chronic kidney disease (CKD) [6]. As a result, the most effective treatments involve addressing and managing underlying disease. Early data suggests that implementation of this strategy shows AF symptom severity and mortality have decreased while quality of life has increased [7]. The CRF-AF association is an area that currently lacks randomized control trials (RCTs) and instead relies mostly on observational data. However, the limited research available is obscured by multivariate influences of exercise intensity, gender, race, and—in many cases—lack of objective data. Nevertheless, investigators have developed several hypotheses about these results to isolate and disentangle this multifactorial relationship between exercise and AF [8].

In this review, we provide an update on the impact of exercise on AF with a special focus on how these differences are stratified by race and gender. We will cover potential mechanisms behind these findings yet also comment on the available research’s limitations. Lastly, we will offer a brief overview of the role of modifying PA in the treatment of patients with preexisting AF.

## 2. Methods

We performed a narrative review investigating the role of CRF in AF, especially as it relates to race and gender. We conducted our search using the PubMed online database using relevant articles published after the year 1998 using keywords “cardiorespiratory fitness”, “atrial fibrillation”, “exercise”, “physical activity”, “race”, “gender”, and “pathophysiology”. A combination of these respective keywords with Boolean operators “OR” and “AND” was utilized, along with medical subject headings (MeSH) terms and their respective synonyms. Only articles written in English were included. The articles’ merit, limitations, applicability, and conclusions were evaluated by the authors for inclusion into this review.

## 3. Atrial Fibrillation Epidemiology and Risk Factor Modification by Enhancing Cardiorespiratory Fitness 

The epidemiology of CRF and AF involves a complex interplay between various factors, some of which being age, race, and gender. Current estimations show that 1 in 4 people are at risk of developing AF at some point in their life [8]. The strongest risk factor for developing AF is increasing age. Increased AF incidence with age is believed to result from cumulative exposure to longstanding, subclinical inflammation from other diseases or environmental influences [9]. Similar to older populations, diagnosis of AF in individuals younger than 65 years is becoming more prevalent [3]. In these age groups, the most effective method in combating AF incidence is through reducing common AF risk factors. One approach to do so is performing regular mild to moderate PA of ≥150 minutes per week as recommended by established guidelines [3]. The physical inactivity of a sedentary lifestyle is strongly corelated with increased risk of developing AF [3]. The precise mechanism(s) involved in the CRF-related mitigation of AF incidence are not completely understood. It is likely that the favorable modulatory effects of PA on known risk factors—such as DM, HTN, and obesity—contribute substantially to the reduction in AF incidence.

Indeed, exercise has been shown to be a suitable intervention to improve AF clinical outcomes by reducing incident risk of related cardiovascular conditions in patients with AF [10, 11, 12, 13, 14, 15, 16]. According to the latest Center for Disease Control estimates, over 38.4 million Americans suffer from DM, and 34.3% of Americans diagnosed with DM are considered physically inactive [17]. In their analysis of over 20 cohort studies, Warburton *et al*. [18] (2010) determined that 84% of participants exhibited a dose-response relationship with even minor positive changes in PA leading to significant reductions in DM incidence [19]. The prevalence of AF is over twice as likely in individuals with DM compared to those without DM [20]. Similarly, roughly 120 million Americans live with HTN [21] and, consequently, face a 1.8-fold higher likelihood of developing AF [22]. Individuals who partake in exercise training experience decreased systolic and diastolic blood pressures, and in some cases, a HTN diagnosis can even become reversible [23, 24]. Nevertheless, it is important to note that the effect of decreased blood pressure following exercise is transient, thus emphasizing the importance for PA to be a regular practice [23]. Sedentary individuals with chronic HTN develop cardiovascular disease at a rate that is 2-fold greater than those without HTN [25]. Lastly, obesity follows a similar trajectory to the other mentioned AF risk factors. Over 140 million Americans are considered clinically obese [26], and with every additional point on the body mass index scale, the incidence of AF accordingly increases by 17% [27]. The Norwegian HUNT3 study reported that the relative risk of obese inactive individuals for developing AF was 1.96, whereas this ratio decreased to 1.53 for obese individuals who engaged in regular PA [28]. Each of these three comorbidities exhibit interconnected relationships and propagate each other’s progression, thus causing unfavorable outcomes in individuals with or at-risk for cardiovascular diseases such as AF [29]. Addressing one comorbidity with exercise may positively impact others and reduce future incidence of AF, especially in select groups who are most at risk for these risk factors and thus AF.

Evidence indicates that AF does not affect every population equally [8]. The lifetime risk is greater than 30% in White individuals, 20% in Black individuals, and 15% in Chinese individuals [30]. Despite White groups having the highest risk for AF, disease severity is far worse in diverse racial populations [31]. For example, while the prevalence of AF is significantly higher among White individuals, the mortality rate is higher in Black patients [32, 33]. One study cites a more accurate clinical process for diagnosing AF in the White populations as a potential explanation for this finding, as Black patients received diagnoses later in the course of AF progression as exacerbations led to severe symptoms [34]. In contrast, White patients were diagnosed at earlier stages, enabling them to be better prepared to mitigate the risk of experiencing severe symptoms. Interestingly, the disparity in early diagnosis was corrected with an ambulatory electrocardiogram device in this cohort, which potentially indicates a true prevalence for Black groups closer to their White counterparts [34]. Moreover, in those who have been successfully diagnosed with AF, Black patients also suffer stroke at a higher rate [34]. This elevated stroke risk holds true in Hispanic populations as well, as Hispanics to have a 2.46 times increased likelihood of stroke recurrence compared to non-Hispanic White patients [35]. Ultimately, the increased rates of stroke occurrence in the non-White populations can be combatted through a more homogenous system for diagnosing AF across all races.

Given the high prevalence of AF in the population, it is imperative to create solutions to prevent deaths and other complications. Certain racial groups may be at higher risk for greater disease burden and thus require increased attention for diagnosis and management. Patients with obesity, HTN, and DM are at elevated risk and particularly susceptible, and enhancing CRF may be an apt way to decrease AF incidence. Evidence in the last few decades has corroborated the utility of this lifestyle change of increasing physical activity, yet more questions arise when discussing the safest possible way of doing so.

## 4. Explaining the Exercise Paradox in the Development of Atrial Fibrillation 

Despite the obvious benefits of exercise on mortality and morbidity from cardiovascular disease, recent evidence has provided mixed results for the previously well-defined phenomenon that associates high-intensity PA and AF. This ‘exercise paradox’ was first reported over two decades ago with the observation that found increased risk for AF in middle and older-aged veteran runners compared to non-athlete controls [36]. Since this initial finding, large cohort studies have established this interesting dynamic between high fitness levels and the development of AF, especially in endurance-based exercise. Andersen *et al*. [37] (2013) showed those athletes who completed the most cross-country skiing races and had the fastest finishing times were more susceptible to AF in the future, albeit only in younger cohorts. A follow-up study in 208,654 Swedish skiers demonstrated a similarly powerful association of exercise with AF incidence, yet these results were stratified by gender [38]. In cyclists, increased AF diagnosis was observed in those athletes who had greater heart structure remodeling; both endpoints were witnessed more frequently in individuals with increasing cycling years compared to age-matched controls [39]. This suggests the effect of cumulative exercise years at older ages may increase arrythmia risk, which was replicated in a later study [40]. Interestingly, former strength-trained athletes in the National Football League (NFL) exhibited higher rates of AF than age and race-matched controls [41], which together asserts that AF is linked to extreme, sustained levels of exercise irrespective of the sport type [42]. Dose-dependent relationships with exercise and AF incidence are also depicted in the general population, with every 1000 steps being associated with small, incremental increases in risk [43]. Overall, the connection between high-intensity training and increased AF occurrence has been upheld across the years by cohort studies of younger and mostly middle-aged athletes, and other investigations into older athletic populations have echoed similar sentiments [44, 45]. This latter point however may be confounded by an increased prevalence of the aforementioned cardiovascular risk factors [46]. Nevertheless, meta-analyses of this association show less certainty. Using many of the same studies in their meta-analyses, Mishima *et al*. [47] (2021) and Kunutsor *et al*. [48] (2021) revealed no increased AF risk with high-intensity exercise; however, the latter study’s findings were segregated by gender—with more pronounced AF risk at higher PA for men but less for women—and were limited by a lack of studies in elderly populations. Even still, analysis of 13 athlete cohort studies with 70,478 participants illustrated markedly elevated risk for AF compared to controls (Odds ratio: 2.46; 95% confidence interval (CI) 1.73 to 3.51; *p* < 0.001, Z = 4.97) [49]. The researchers also included several elderly athlete cohorts with subsequent subgroup analysis of age failing to show significantly increased risk. The most recent data seems to corroborate earlier findings of increased AF incidence in high-intensity younger to middle-aged athletes. Similar to the findings in older athletes, however, this has not yet been upheld in broader population-spanning investigations.

In contrast to the exercise paradox, the most recent large-population studies affirm that incorporating mild-to-moderate exercise helps reduce the incidence of AF. In previously healthy patients, observational studies from a large cohort of over 500,000 middle to older aged-individuals in the United Kingdom (UK) have shown reduced incidence of AF in patients with metabolic equivalent (MET)-minutes per week greater than 1500, high net oxygen consumption (${\mathrm{VO}}_{2}$), and increased daily accelerometer-derived global PA [50, 51, 52]. Interestingly, a novel finding from the UK group demonstrated that concentrating exercise to two days a week compared to spreading activity throughout the week decreased incident AF risk at similar [53]. Taken together, these newer findings are consistent with similar studies in other populations and show that AF risk is inversely associated with meeting guideline-directed exercise requirements in younger and middle-aged persons [54, 55, 56, 57]. This was also found to be true for elderly patients and may be due to increased importance of risk factor modification or decreased overall exercise intensity as one ages [58, 59, 60]. Many of the aforementioned studies utilized objective, accelerometer-based tracking of PA, but others incorporated more subjective manners of data collection using questionnaires such as the international physical activity questionnaire (IPAQ) [50, 51, 54, 57, 61]. While the latter results are partially limited by the bias inherent to patient-reported outcomes, meta-analyses with millions of participants still demonstrate the value of risk stratification using this metric by showing decreased AF incidence stratified by different levels of reported activity [47, 48, 62]. Nevertheless, it is important to note the observational nature of these large studies, with direct causality and preventability unable to be determined. The most recent approximation was a prospective cohort of elderly adults that associated greater physical decline, grip strength, walk distance, and walk time with incident AF [63].

Overall, the newest research on exercise and incident AF utilizing wide-spanning cohorts maintain the assertion that those younger to middle-aged individuals achieving high levels of PA are at increased risk of acquiring AF, especially in populations comprised of athletes, but in the general and elderly athlete population, the results are unclear. Patients who adhere to guideline-recommended exercise standards, however, are more likely to confer protective benefits from AF. These data should be cautiously interpreted due to their liberal use of subjective data, being largely retrospective, and observational nature. More definitive statements about the relationship between CRF and incident AF will require more stringent study designs, with hope to the future in the Master@Heart (NCT03711539) [64] and Prospective Athlete’s Heart Study (NCT05164328) [65] prospective cohorts. Moreover, the international NEXAF Detraining study (NCT04991337) will investigate how reduced exercise levels impact management in endurance athletes with preexisting AF, thus elucidating further any possible connection between exercise levels and subsequent AF burden [66]. Despite the great work that has been accomplished in observing this relationship, the current research landscape often reports the effect of varying levels of PA on AF incidence without commenting on known socioeconomic risk factors that affect the development of AF, including gender and race.

## 5. Gender Differences in Cardiorespiratory Fitness on Incident Atrial Fibrillation 

Incorporating regular exercise for the prevention and management of AF is important for both men and women, but the differential impact of gender should be considered before making recommendations. Both at rest and in response to exercise, notable differences exist in the structure and function of a female versus male hearts [67, 68, 69, 70]. Accordingly, the guidelines that utilize CRF in the diagnosis, treatment, and prevention of AF should ideally be developed with this sex-specific intention [71]. However, the available evidence from the most impactful cohorts involving the effect of exercise on AF in the general population predominantly consist of most—if not all—male participants or reports no gender-mediated distinctions [13, 15, 27, 56]. Even with this limitation in mind, recent data examining clinically-relevant differences in AF incidence according to sex with varying levels of exercise suggests increased benefit in women compared to men [72]. Using the large UK Biobank cohort, Elliott *et al*. [50] (2020) found that performing above guideline-recommended exercise levels up to 5000 MET-min/week exhibited increasing risk reduction in AF incidence in women, but not in men. In this sample, vigorous exercise in men led to a 12% increased risk of developing AF (hazard ratio: 1.12, 95% CI 1.01–1.25), which starkly contrasts with the observed 8–16% decreased risk of AF incidence in women (hazard ratio: 0.80, 95% CI 0.66–0.97). Similar results were found in a well-represented Korean population [54] and the Women’s Health Initiative Observation Study [73]; however, no differences were noted in the smaller Atherosclerosis Risk in Communities study cohort [74]. Furthermore, multiple meta-analyses have supported the hypothesis that women have increased exercise tolerance compared to men when stratifying AF risk [48, 62, 75], although the newest data acknowledges potential confounding biases given the observational nature of the available evidence [76]. While men seem to have a greater prevalence of AF risk factors than women, women who are ultimately diagnosed with AF are more likely to have HTN, a positive smoking history, and hyperlipidemia than their male counterparts [77]. Therefore, investigators have explored this relationship and cited improved mitigation of risk factors such as blood pressure and body-mass index using exercise as the key drivers of decreased AF incidence in women versus men [78, 79]. Taken together, the available evidence cautiously suggests that women can safely pursue PA at higher levels than men without increasing risk of developing AF, which has important clinical implications when counseling female patients on how best to prevent future disease by improving their CRF.

In exercising athletes, AF risk differs between men and women. Consistent with previous observations in the general population, AF studies in athletes are primarily performed in predominantly male populations [36, 37, 39, 41] which has been acknowledged in subsequent meta-analyses [49]. Many factors have been implicated in the lack of studies in women athletes, such as historically low participation in sporting events, lack of overall AF events, shorter race distances, and decreased cumulative sports exposure [80, 81]. As summarized previously in those research efforts, well-conditioned men seem to suffer an increased risk of developing AF [36, 37, 38, 39, 41]. At this point in the literature devoted to female athletes, however, therein remains insufficient data to make the same conclusions with certainty. As a follow-up to the first report of increased AF risk in female athletes [81], Svedberg *et al*. [38] (2019) followed a cohort of competitive skiers (126,342 men and 82,312 women) and demonstrated decreased AF incidence in women at all levels of activity and athletic performance, but increased AF incidence in men who participated in more races and performed better compared to non-skier-matched controls. Contrarily, a recent research letter from Myrstad *et al*. [82] (2024) prospectively examined skiers from the Tromsø Study cohort and showed similar risk from cumulative exercise exposure, echoing previous findings seen before in only men. Another study in Sweden followed women athletes who had previously completed marathons and cycling at elite levels was the first of its kind to capture an increased risk of acquiring AF in the high-endurance athlete cohort (hazard ratio: 2.56; 95% CI 1.22 to 5.37) [83]. However, this latter investigation only had 228 individuals in the female athlete group, echoing previous challenges of surveying women athlete populations [80, 81]. Overall, more studies with greater inclusion of women athletes are needed to more appropriately guide AF consideration, and the upcoming Prospective Athlete’s Heart Study is actively recruiting men and women athletes and will help address this gap [65].

In summary, disparate levels of AF incidence are not only visualized across varying CRF and PA levels, but also through the influences of gender (Table 1, Ref. [13, 15, 27, 36, 37, 38, 39, 41, 44, 50, 51, 53, 54, 56, 57, 58, 72, 73, 74, 79, 81, 82, 83]). The current literature implicates high-intensity exercise in men with increased AF risk, yet the same does not hold true for women, although with more limited evidence. In both athlete and non-athlete populations, women are generally able to tolerate even extreme levels of PA without incurring the same potential of developing AF. Mechanistically, this could be due to simply a lack of available data or from differences in anatomy and responses to exercise, the latter of which will be explored in detail later in this review. Moving forward, special efforts should be made to include well-represented populations to better elucidate gender’s part in the relationship between AF and CRF.

**Table 1.** **Examining the relationship between physical activity and atrial fibrillation in men versus women**.

	Men	Women
Low-to-moderate intensity exercise	Decreased AF incidence	Decreased AF incidence
High-intensity exercise	Increased AF incidence	Decreased AF incidence*
Potential mechanisms	Differences in cardiac anatomy, risk factor reduction, and responses to exercise by sex	
Landmark studies in non-athletes by predominant gender representation	Kokkinos *et al*. (2008) [13]	Mozaffarian *et al*. (2008) [58]
	Kokkinos *et al*. (2017) [15]	Azarbal *et al*. (2014) [73]
	Khan *et al*. (2015) [56]	Tikkanen *et al*. (2018) [51] ‡
	Morseth *et al*. (2016) [57] ‡	Garnvik *et al*. (2019) [72]
	Kamil-Rosenberg *et al*. (2020) [27] ‡	Jin *et al*. (2019) [54] ‡
		Elliott *et al*. (2020) [50] ‡
		Fletcher *et al*. (2022) [74] ‡
		Khurshid *et al*. (2023) [53]
		Sharashova (2023) [79] ‡
Landmark studies in athletes by predominant gender representation	Karjalainen *et al*. (1998) [36]	Myrstad *et al*. (2015) [81]
	Baldesberger *et al*. (2008) [39]	Myrstad *et al*. (2015) [81]
	Andersen *et al*. (2013) [37] ‡	Drca *et al*. (2023) [83]
	Myrstad *et al*. (2014) [44]	Myrstad *et al*. (2024) [82] ‡
	Aagaard *et al*. (2019) [41]	
	Svedberg *et al*. (2019) [38] ‡	
Recommendation	In healthy men:	In healthy women:
	Perform low-to-moderate PA, uncertain benefit for high-intensity exercise and potential for harm	Exercise without intensity restrictions,* but more studies are needed in athletic populations

AF, atrial fibrillation; PA, physical activity. *Based on the limited data available in the general population. ‡Includes both men and women.

## 6. The Influence of Race on Atrial Fibrillation Prevention with Physical Activity 

Importantly, the impact of PA on AF risk can be observed through the sociocultural lens of race. Despite an increased prevalence of known AF risk factors and subsequently worse outcomes in Hispanic and Black populations [84], AF has a higher incidence in White individuals [85, 86]. Many causes of this association have been hypothesized, including genetic predisposition due key susceptibility loci present in individuals of European descent but absent in other ethnicities [87]. While the true driver behind this observation is likely multifactorial, some believe this may be due to increased diagnosis, awareness, and other lifestyle factors in White patients compared to non-Whites [34, 88, 89]. Even still, the role of exercise in reducing incident AF in different racial communities remains unclear. Many of the aforementioned large observational studies are performed on mostly non-diverse populations, and they control for race in their findings using statistical predictive models or fail to comment on any potential influence [50, 51, 52, 53, 54, 56]. Of the recent major studies that have included race, exercise affects incident AF risk in all ethnic groups in similar fashions [74, 90]. This is consistent with the previous reports comparing all racial populations. Nevertheless, therein remains a great need for more work that explores racial differences, as the current evidence of PA’s influence on AF is largely based on populations that are homogenous, thus limiting comparison to other non-represented persons. In particular with athletes, the hallmark studies in the field have been examined in majorly White participants [36, 37, 38, 39, 41]. We located one single study with sufficient representation of non-White subjects. This study was performed in former NFL players and showed that Black race was independently associated with decreased AF risk in high-strength training athletes, a finding that warrants further investigation in diverse athletes in different sport types [41].

Even with an apparent lack of studies specifically analyzing this relationship, others have investigated ethnicity’s role on the several established measures of CRF that factor into the larger, more pertinent studies with AF. Some articles have reported racial differences in ${\mathrm{VO}}_{2}$ max between White and Black races due to unique mitochondrial and energy expenditure characteristics, but these findings are limited due to the small sample size and lack of correction for confounding variables [91, 92, 93]. This has yet to be correlated with large scale, prospective clinical investigations in patients at risk for AF. However, other research taking risk factors into consideration revealed substantial differences in ${\mathrm{VO}}_{2}$ max in non-Hispanic White, Hispanic, and Asian groups [94]. Similarly, Black men had increased peak systolic blood pressure and decreased peak ${\mathrm{VO}}_{2}$ max compared to their White counterparts in a cohort study of almost 6000 participants undergoing exercise testing [95]. Moreover, disparities exist in average weekly METs between racial groups. Bazargan-Hejazi *et al*. [96] (2017) demonstrated that African Americans were less likely to achieve the recommended 150 minutes of directly observed 150 MET minutes per week than Caucasians and Hispanics. South Asian adults in the United States are as likely as White adults to engage in sufficient weekly aerobic exercise (150–300 MET minutes per week) but less likely to reap the benefits of high-intensity exercise of over 300 MET minutes per week [97]. Although many such studies utilize direct measurements of PA, caution must be exercised in their interpretation due to confounding variables of leisure PA versus work PA that contributes to inequality in measurement [98]. Even still, while no racial disparities were found in predicting cardiovascular and all-cause mortality using exercise status, overall CRF is still relevant in determining prognosis across all ethnic groups [99, 100].

Indeed, while race has not yet been shown to definitively stratify incident AF risk in exercising individuals, non-White populations remain more susceptible to predisposing, known predictors of AF. In all areas of cardiovascular health, efforts to reduce development of and death from cardiovascular disease have been disproportionately unsuccessful in specific racial demographics, especially African Americans [101]. The cause of this inequality is complicated, with evidence emerging that reveals exercise as a varied effect modifier on known AF risk factors in diverse ethnic groups [102]. Therefore, integrating CRF-improving programs must consider the needs of different racial communities to prevent AF incidence and other cardiovascular comorbidity. To this point, no prospective studies have been specifically designed to capture race-sensitive interventions to prevent incident AF. However, future studies can look towards previously successful, cardiovascular-focused interventions to decrease abundant AF risk factors common to these vulnerable groups. Most population-based clinical trials have focused on Black individuals and encouraged exercise through exercise sessions, educational classes, social cohesion, and smart-phone applications to favorably improve short and long-term exercise adherence [103, 104, 105, 106]. Studies in Asian American, Native Hawaiian, and South Asian American groups integrated culturally-specific teaching and community sessions to promote PA and improve AF risk factors of high blood pressure, DM, and obesity [107, 108, 109]. Given the lack of statistically significant efficacy of such programs in adult Latino populations, additional investigations are ongoing to identify successful PA-improving strategies in this community [110].

**Fig. 1.**
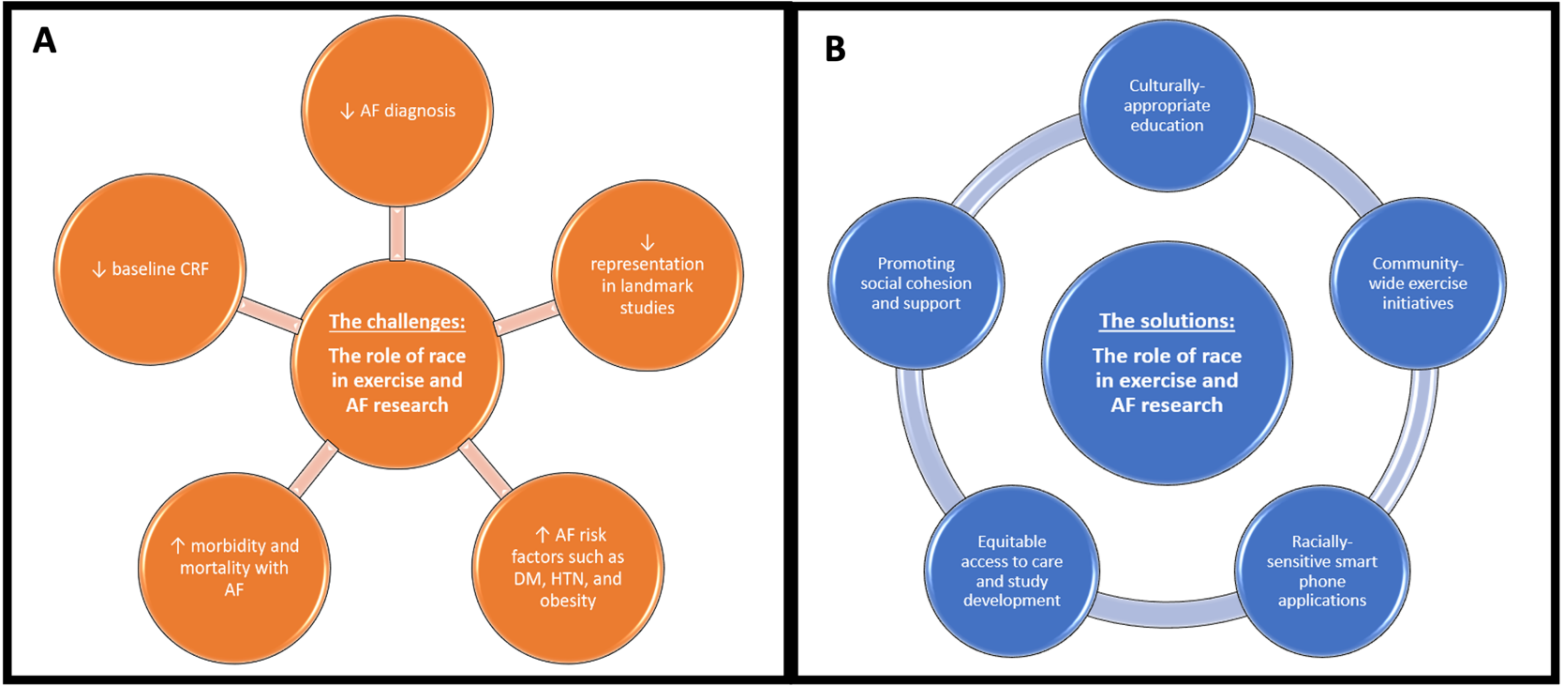
**The obstacles and solutions observed in the investigations examining atrial fibrillation (AF) and exercise.** (A) Issues related to performing research in minority groups and the unique challenges they face. (B) Successful initiatives in vulnerable racial populations. CRF, cardiorespiratory fitness; DM, diabetes mellitus; HTN, hypertension.

Overall, the evidence surrounding CRF differences and interventions for AF in diverse populations is sparse at best. For the translational advancement of the field, it is vital for future research to include and consciously stratify individuals from various ethnic backgrounds during study design and data collection. An understanding of these unique challenges (Fig. 1A) has preceded creative solutions to target these vulnerable groups and permitted subsequent research on the effect PA on AF (Fig. 1B). Therefore, developing culturally specific interventions should be considered when hoping for the best success in preventing incident AF with exercise.

## 7. The Risks and Benefits of Improved Cardiorespiratory Fitness on Atrial Fibrillation Pathophysiology 

**Fig. 2.**
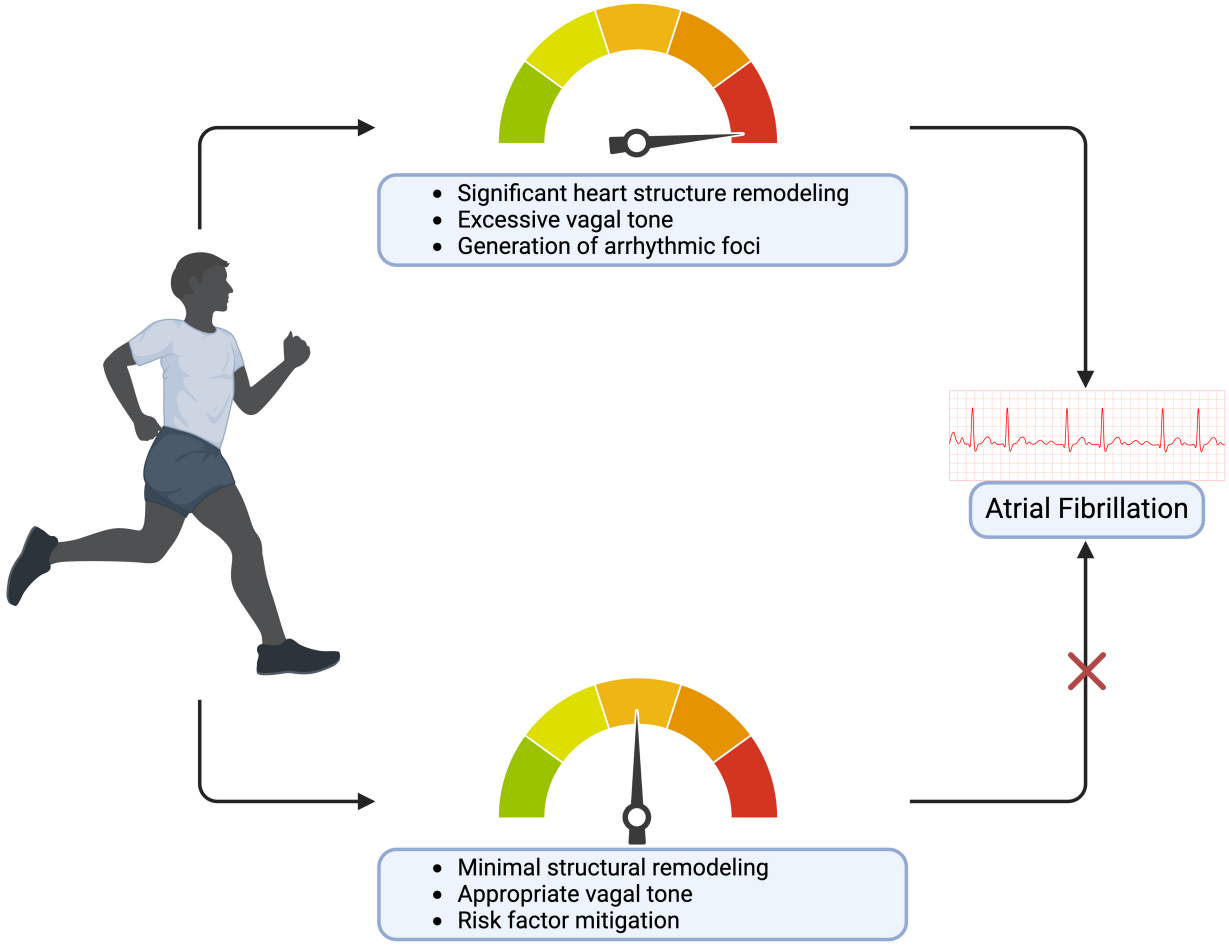
**The risks and benefits of varying exercise levels on atrial fibrillation.** Created on https://www.biorender.com/.

Exercise is often prescribed by providers to patients at high-risk for AF, but before doing so, it is important to recognize how the effects of PA on heart structure and function can become deleterious. Clinical data suggests that high-intensity endurance athletes are more prone to developing AF than others who limit exercise to moderate levels [111, 112, 113]. The pathogenesis behind this paradox can first be explained by examining the root causes of AF—the formation of ectopic foci in the heart by arrhythmic triggers. This unsynchronized firing leads to AF and begets structural changes. Specifically, increased vagal tone, atrial inflammation, fibrosis, cardiac remodeling, and left atrium enlargement are known causes of AF and may trigger these ectopic foci in high-performance athletes [114]. Subsequently, these compounding structural and electrical adaptations from exercise result in the development of AF. Furthermore, a training athlete’s heart adapts to high-intensity exercise by increasing left ventricular diameter and volume, which is believed to subsequently increase vagal tone [115]. Trivedi *et al*. [116] (2020) examined athletes with AF and observed preserved left ventricular diastolic parameters with enlarged right atria compared to control. Instead, structural changes in nonathletes with AF had diastolic dysfunction and reduced left atrial strain, indicating a difference in pathophysiological mechanism as a result of exercise [116]. Differences in left and right heart structure and function were also observed according to athletic status and presence or absence of AF in older patients [117, 118, 119]. In a state of profound dominance of vagal tone, the parasympathetic nervous system is heightened, and sympathetic activity is diminished. This represents a sharp departure from the extreme sympathetic surge at the height of PA, with the increased vagal tone at rest disrupting the balance between the two systems. For this reason, autonomic imbalance has been hypothesized to play a significant role in the onset of AF, yet more studies are needed to confirm this belief [120]. Additionally, evidence suggests that intensity and duration of exercise is proportional to heart structure remodeling, which in turn increases one’s susceptibility to AF [121]. This pathogenesis has been hypothesized mainly for aerobic exercise, with little data available to explain one finding of increased AF risk in primarily resistance-trained athletes [41]. Taken together, this could explain how the exercise paradox in highly active populations results in increased AF incidence (Fig. 2).

Current investigations implicate high-intensity training in the development of AF in men, but not in women, and variances in heart anatomy may be able to partially clarify gender’s differential effect of AF incidence stratified by exercise. Early research discovered that female endurance athletes have lower cardiac output, diastolic filling rate, left ventricular ejection rate, and a higher maximum arteriovenous global oxygen delivery compared to their male endurance athlete counterparts [68]. This suggests that under high-intensity exercise, the female heart experiences less sympathetic escalation and thereby less compensatory parasympathetic innervation, which together functions to prevent increased vagal tone and overall decreases the likelihood of incident AF [112, 122]. Conversely, male endurance athletes have significantly larger left atrial volumes, which create the anatomical substrates that propagate the re-entry mechanisms at the heart of AF [69]. Accordingly, male endurance athletes undergo more severe cardiac remodeling that exacerbates AF incidence when compared to females. Left ventricular characteristics is another commonly used metric for diagnosing AF, and one study comparing the left ventricle geometry of male and female athletes across various sports found that female athletes undergo restructuring less frequently than male athletes [70]. This supports the long-standing theory that higher activity levels lead to more heart remodeling.

**Fig. 3.**
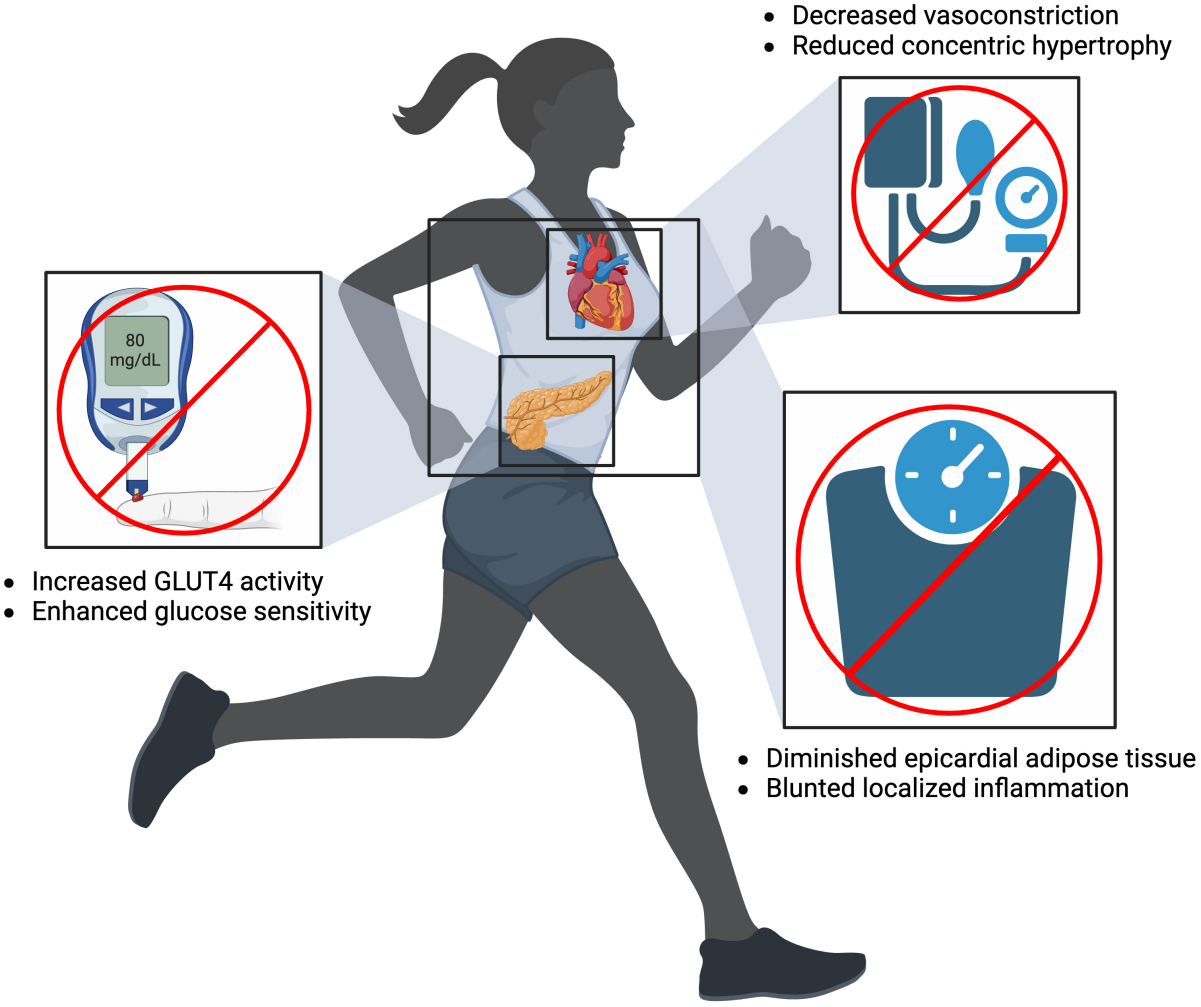
**Mitigating atrial fibrillation risk factors of diabetes mellitus (left), hypertension (top right), and obesity (bottom right) by increasing physical activity.** Created on https://www.biorender.com/. GLUT4, glucose transporter type 4.

Improving PA is a particularly effective method of mitigating AF risk factor development. Common risk factors for AF have been shown to induce structural and electrophysiological abnormalities by creating conditions for aberrant reentry and focal ectopic activity [4]. For example, physical inactivity is a common cause of HTN and a known risk factor of AF. Sedentary lifestyles relegate the body to a state of decreased metabolic demands. This leads to the closure of precapillary sphincters and vascular beds to then decrease nitrous oxide production in endothelial cells, which promotes vasoconstriction and subsequent HTN [123]. Over time, HTN promotes concentric hypertrophy, which eventually leads to AF due to maladaptive heart anatomy. Encouraging exercise reverses this cascade of events and thus prevents the development of HTN. Increasing PA also prevents AF by combating obesity. Similar to HTN, obesity is associated with poor CRF. Low metabolic demands equate to decreased glucose usage and increased fat storage everywhere in the body, including the heart. Epicardial adipose tissue (EAT) specifically acts as a direct trigger for AF by increasing local inflammation via inflammatory cytokines (interleukin (IL)-1β, IL-6, and tumor necrosis factor (TNF)) due to close proximity to the heart muscle [124, 125, 126]. Individuals participating in regular PA have markedly decreased EAT, with one study finding that endurance and resistance training correlates with a 32% decrease in EAT [127]. Lastly, exercise plays a role in DM pathogenesis by enhancing glucose homeostasis [128]. Observational studies suggest regular PA increases activity of glucose transporter type 4 (GLUT4)—the transporter for glucose within muscle cells—to improve insulin sensitivity and glycemic control [129, 130]. This effect has clinical implications for patients, as doing as little as 5–7 hours of exercise per week results in a 55% decreased risk of developing DM [128]. Evidentially, each of these AF risk factors promote negative metabolic and cardiovascular consequences on the body, which exacerbates AF development. Enhancing CRF is therefore a vital tool in combating these risk factors and, as a result, AF incidence (Fig. 3).

In conclusion, those who participate in regular, moderate PA have displayed the greatest outcomes in AF prevention and management by reaping the most benefit from exercise without experiencing its pitfalls. Moderate PA promotes strengthening of the heart and mitigation of AF risk factors without skewing the autonomic nervous system balance. High-intensity exercise, however, leads to increased vagal tone and changes in heart anatomy that serve as a nidus for AF. Hence, increasing CRF with exercise to the extent that maintains heart health and autonomic nervous system balance will promote normal heart rhythm.

## 8. Exercise in Patients with Preexisting Atrial Fibrillation

Contrary to the debate circulating exercise and AF primary prevention, the evidence supporting CRF enhancement in patients with AF is much more concrete. The majority of studies involving exercise in patients with AF are observational and associate increased PA with decreased major adverse cardiovascular events, improved mortality, increased tolerance of damaging comorbidities, better ablation outcomes, and reduced symptoms [131, 132, 133, 134, 135, 136, 137]. While these populations provide important relationship data to the field, the RCTs that explore this dynamic permit even stronger conclusions to be made: Exercise remains a cornerstone for treatment of preexisting AF patients. Some research in the area have targeted CRF and quality of life parameters as clinical outcomes, as patients with AF who perform aerobic exercise demonstrate better symptom control, physical functioning, exercise capacity, handgrip strength, 6-minute walk tests, and peak ${\mathrm{VO}}_{2}$ compared to control [138, 139, 140, 141, 142, 143, 144]. Other trials examine common measures of AF treatment such as decreased AF recurrence or burden and increased freedom from AF, all of which have been successfully achieved [142, 143]. To summarize, a comprehensive meta-analysis of 13 RCTs confirmed the utility of exercise in patients with paroxysmal or persistent AF by noting improved CRF, quality of life, and AF recurrence, but not AF burden when all studies were included [145].

As previously discussed, patients without AF may face increased risk at higher exercise levels, yet this contrasts with the noted benefits from high-intensity training in patients with preexisting AF. This may be due to short average follow-up times of 12–16 weeks, the high-reward PA benefits in sedentary individuals, increased risk factor burden and subsequent modification, and/or a lack of continuous exposure over cumulative exercise years. Therefore, given the evidence surrounding the exercise paradox, concern is warranted in prescribing the appropriate amount of exercise in AF. Just the same, Skielboe *et al*. (2017) [146] asserted no difference in AF burden or hospitalizations due to AF in patients randomized to high or low-intensity with a 1-year follow-up. However, this lies contradictory to the dose-dependent decreased AF risk seen in rising exercise levels depicted in the CARDIO-FIT prospective cohort, which creates further uncertainty [147]. The upcoming NEXAF Detraining study should help to clarify some of these observations [66]. Increasingly, many of the previous and upcoming investigative trials choose exercise programs and risk factor modification strategies to accomplish weight reduction endpoints and treat AF, one of the most notable being the LEGACY cohort [148]. In combination with diet modification and lifestyle counseling, the LEGACY trial examined the long-term effect of low-to-moderate intensity exercise on patients with paroxysmal and persistent AF and found decreasing AF burden and arrhythmia-free survival in the group with the most weight loss, which was maintained over the course of several years [148]. The RACE 3 trial similarly targeted known AF risk factors through a combination of pharmacological and exercise interventions and showed improved restoration of sinus rhythm after 1-year [149]. While the results of these RCTs are promising, the most impactful trials cited by the 2023 guidelines on the diagnosis and management of AF [3] feature key limitations within the makeup of study participants. Many trials examining AF treatment with PA have very limited representation of women [138, 140, 141, 142, 144, 148, 149, 150]. Similarly problematic, the racial demographic of those people included is almost universally underreported or, as seen in the one trial, compromised of strictly White individuals (approximately 97% in all groups) [146].

While further details are outside the scope of this review, it is important for cardiologists to encourage regular, guideline-recommended PA in patients with all forms of AF. Multiple RCTs demonstrate favorable short and long-term outcomes and significant improvements in symptomology. There is still a great need for improved representation of diverse racial groups and female patients in current investigations to help understand the unique characteristics of each population. Special considerations should also be made for patients with differing comorbidity burden [151], which modulates the degree a patient should exercise for maximum benefit. The results and makeups of the most significant RCTs studying CRF and AF are depicted in Table 2 (Ref. [138, 139, 140, 141, 142, 143, 144, 146, 149, 150]).

**Table 2.** **Recent randomized controlled trials and results investigating exercise in patients with atrial fibrillation**.

Trial	Population	Intervention	Notable results	Gender demographic (male)	Racial makeup
Hegbom *et al*. (2007) [138]	Chronic AF (n = 28)	Aerobic and muscle strengthening exercise program compared to no training	- Improved SF-36 and SSCL in EG	Treatment: 100%	Not reported
			- Improved exercise capacity and perceived exertion in both groups	Control: 87%	
Osbak *et al*. (2011) [139]	Permanent AF (n = 51)	Aerobic exercise program compared to no training	- Improved 6MWT, exercise capacity, MLHF-Q, and SF-36 in EG	Treatment: 43%	Not reported
				Control: 43%	
${\mathrm{CopenHeart}}_{R\,F\,A}$ [140]	Paroxysmal or persistent AF undergoing ablation (n = 210)	Cardiac rehabilitation with exercise training compared to usual care	- Improved peak ${\mathrm{VO}}_{2}$ max in EG	Treatment: 74%	Not reported
			- No difference in SF-36 between both	Control: 77%	
			- Increased non-serious adverse events in EG (16 versus 7, *p* = 0.047)		
Malmo *et al*. (2016) [141]	Symptomatic, non-persistent AF (n = 51)	Aerobic interval training compared to usual exercise habits	- Improved mean time in AF, SF-36, SSCL, peak ${\mathrm{VO}}_{2}$ max, LA and LVEF, cardioversions, and hospitalizations in EG	Treatment: 77%	Not reported
				Control: 88%	
Skielboe *et al*. (2017) [146]	Paroxysmal or persistent AF	High-intensity or low-intensity aerobic exercise	- No difference in AF burden and hospitalization	High-intensity: 59%	97% White in both groups
	(n = 70)		- Both groups improved peak ${\mathrm{VO}}_{2}$ max with no difference	Low-intensity: 58%	
Rienstra *et al*. (2018) [149]	Early persistent AF and mild-to-moderate HF (n = 245)	Aggressive risk factor modification with pharmacology, lifestyle counseling, and cardiac rehabilitation	- Increased percentage of restored sinus rhythm in EG	Targeted therapy: 79%	Not reported
			- Improved risk factor control* in EG	Conventional therapy: 79%	
			- Similar LVEF improvements and hospitalizations due to AF in both groups		
Kato *et al*. (2019) [142]	Persistent AF undergoing ablation (n = 6)	Cardiac rehabilitation with exercise training compared to usual care	- Improved 6MWT, handgrip and leg strength, and LVEF in EG	Treatment: 71%	Not reported
			- No difference in AF recurrence	Control: 90%	
ACTIVE-AF [143]	Symptomatic paroxysmal or persistent AF (n = 120)	Patient-tailored exercise program compared to usual care	- Improved freedom from AF after 12 months and AFSS after 6 and 12 moths in EG	Treatment: 58%	Not reported
			- Both groups increased peak ${\mathrm{VO}}_{2}$max	Control: 57%	
Alves *et al*. (2022) [144]	HFrEF with permanent AF (n = 26)	Aerobic and muscle strengthening exercise program compared to no training	- Improved peak ${\mathrm{VO}}_{2}$ max, MLHF-Q, ventilation slope per minute/${\mathrm{CO}}_{2}$ production with exercise in EG	Treatment: 100%	Not reported
			- Decreased resting HR, recovery HR in EG	Control: 100%	
			- Increased LVEF and structural morphology in EG		
Reed *et al*. (2022) [150]	Persistent or permanent AF (n = 86)	High-intensity interval training compared to moderate-to-high intensity continuous training	- No difference 6MWT, SF-35, AFSS, and time in AF between high-intensity interval training and moderate-to-high intensity continuous training	Treatment: 67%	Not reported
				Control: 65%	

AF, atrial fibrillation; EG, experimental group; SF-36, short-form 36; SSCL, Symptom and Severity Checklist; 6MWT, 6-minute walk test; MLHF-Q, Minnesota Living With Heart Failure Questionnaire; LA, left atrial; LVEF, left ventricular ejection fraction; HFrEF, heart failure with reduced ejection raction; HR, heart rate; AFSS, Atrial Fibrillation Symptom Severity Questionnaire; ${\mathrm{VO}}_{2}$, oxygen consumption; HF, heart failure; *Including changes in blood pressure, weight, body-mass index, and lipid profile.

## 9. Conclusions

In conclusion, the field of CRF in patients with and without AF is rapidly growing but requires some nuance in interpretation of the current evidence. AF is an exacting disease with significant mortality and morbidity. To combat this condition, increasing PA levels can improve several AF risk factors—such as obesity, HTN, and DM—by decreasing vasoconstriction, reducing ETA, and modulating insulin sensitivity. However, recent evidence continues to support the longstanding theory that high-intensity exercise leads to elevated AF incidence, especially in athletes. Extreme levels of physical activity can potentially lead to the formation of harmful triggers, which in turn may cause increased vagal tone resulting in imbalanced sympathetic and parasympathetic activity and subsequent changes in cardiac anatomy. Providers may safely counsel patients of all ages to perform guideline-recommended amounts of low-to-moderate PA; however, prescribing high-intensity exercise remains controversial with unclear benefit at this point. Even still, analysis stratified by sex has revealed decreased AF risk in women following exercise even at extreme levels. This can potentially be explained by differences in hemodynamics and structural responses to exercise by men and women. Nevertheless, most of the research investigating this association between CRF and AF is observational and is therefore inherently limited. For this reason, the optimal level of exercise intensity remains unknown. Similarly, the vast majority of studies include racially homogenous individuals, and as such, forthcoming research should address this need for representation in diverse ethnic groups, specifically Black, Latino, and Asian athlete and non-athlete populations. These groups face inordinately high burden of AF risk factors that can be addressed by enhancing exercise levels, which necessitates follow-up studies to determine the best strategy in doing so. Finally, superior evidence in RCTs in patients with preexisting AF has demonstrated marked improvements in clinically significant AF outcomes and patient quality of life. Despite the current limitations, using CRF to counter the consequences of AF has great promise, and future investigation should expand the discipline by learning more about its place in select racial and gender groups.

## References

[1] Colilla S, Crow A, Petkun W, Singer DE, Simon T, Liu X (2013). Estimates of current and future incidence and prevalence of atrial fibrillation in the U.S. adult population. *The American Journal of Cardiology*.

[2] Tanaka Y, Shah NS, Passman R, Greenland P, Lloyd-Jones DM, Khan SS (2021). Trends in Cardiovascular Mortality Related to Atrial Fibrillation in the United States, 2011 to 2018. *Journal of the American Heart Association*.

[3] Joglar JA, Chung MK, Armbruster AL, Benjamin EJ, Chyou JY, Cronin EM (2024). 2023 ACC/AHA/ACCP/HRS Guideline for the Diagnosis and Management of Atrial Fibrillation: A Report of the American College of Cardiology/American Heart Association Joint Committee on Clinical Practice Guidelines. *Circulation*.

[4] Brandes A, Smit MD, Nguyen BO, Rienstra M, Van Gelder IC (2018). Risk Factor Management in Atrial Fibrillation. *Arrhythmia & Electrophysiology Review*.

[5] Sepehri Shamloo A, Arya A, Dagres N, Hindricks G (2018). Exercise and Atrial Fibrillation: Some Good News and Some Bad News. *Galen Medical Journal*.

[6] Chamberlain AM, Alonso A, Gersh BJ, Manemann SM, Killian JM, Weston SA (2017). Multimorbidity and the risk of hospitalization and death in atrial fibrillation: A population-based study. *American Heart Journal*.

[7] Matei LL, Siliste C, Vinereanu D (2021). Modifiable Risk Factors and Atrial Fibrillation: the Quest for a Personalized Approach. *Maedica*.

[8] Chugh SS, Havmoeller R, Narayanan K, Singh D, Rienstra M, Benjamin EJ (2014). Worldwide epidemiology of atrial fibrillation: a Global Burden of Disease 2010 Study. *Circulation*.

[9] Kornej J, Börschel CS, Benjamin EJ, Schnabel RB (2020). Epidemiology of Atrial Fibrillation in the 21st Century: Novel Methods and New Insights. *Circulation Research*.

[10] Faselis C, Doumas M, Pittaras A, Narayan P, Myers J, Tsimploulis A (2014). Exercise capacity and all-cause mortality in male veterans with hypertension aged ≥ 70 years. *Hypertension*.

[11] Kokkinos P, Myers J (2010). Exercise and physical activity: clinical outcomes and applications. *Circulation*.

[12] Kokkinos P, Myers J, Faselis C, Panagiotakos DB, Doumas M, Pittaras A (2010). Exercise capacity and mortality in older men: a 20-year follow-up study. *Circulation*.

[13] Kokkinos P, Myers J, Kokkinos JP, Pittaras A, Narayan P, Manolis A (2008). Exercise capacity and mortality in black and white men. *Circulation*.

[14] Kokkinos PF, Faselis C, Myers J, Panagiotakos D, Doumas M (2013). Interactive effects of fitness and statin treatment on mortality risk in veterans with dyslipidaemia: a cohort study. *Lancet*.

[15] Kokkinos PF, Faselis C, Myers J, Narayan P, Sui X, Zhang J (2017). Cardiorespiratory Fitness and Incidence of Major Adverse Cardiovascular Events in US Veterans: A Cohort Study. *Mayo Clinic Proceedings*.

[16] Myers J, Kokkinos P, Chan K, Dandekar E, Yilmaz B, Nagare A (2017). Cardiorespiratory Fitness and Reclassification of Risk for Incidence of Heart Failure: The Veterans Exercise Testing Study. *Circulation. Heart Failure*.

[17] CDC (2023). National diabetes statistics report — diabetes —. https://www.cdc.gov/diabetes/data/statistics-report/index.html.

[18] Warburton DE, Charlesworth S, Ivey A, Nettlefold L, Bredin SS (2010). A systematic review of the evidence for Canada’s Physical Activity Guidelines for Adults. *The International Journal of Behavioral Nutrition and Physical Activity*.

[19] Zahalka SJ, Abushamat LA, Scalzo RL, Reusch JEB, Feingold KR, Anawalt B, Blackman MR (2000). *The role of exercise in diabetes*.

[20] PhD LGMP (2022). Association of Atrial Fibrillation with Diabetes Mellitus, High Risk Comorbidities. *Maedica*.

[21] Centers for Disease Control and Prevention Web site. (2023). Facts about hypertension. https://www.cdc.gov/bloodpressure/facts.htm.

[22] Ogunsua AA, Shaikh AY, Ahmed M, McManus DD (2015). Atrial Fibrillation and Hypertension: Mechanistic, Epidemiologic, and Treatment Parallels. *Methodist DeBakey Cardiovascular Journal*.

[23] Carpio-Rivera E, Moncada-Jiménez J, Salazar-Rojas W, Solera-Herrera A (2016). Acute Effects of Exercise on Blood Pressure: A Meta-Analytic Investigation. *Arquivos Brasileiros De Cardiologia*.

[24] Pescatello LS, Kulikowich JM (2001). The aftereffects of dynamic exercise on ambulatory blood pressure. *Medicine and Science in Sports and Exercise*.

[25] Al Khalaf S, Chappell LC, Khashan AS, McCarthy FP, O’Reilly ÉJ (2023). Association Between Chronic Hypertension and the Risk of 12 Cardiovascular Diseases Among Parous Women: The Role of Adverse Pregnancy Outcomes. *Hypertension*.

[26] NIDDK (2021). Overweight & obesity statistics. https://www.niddk.nih.gov/health-information/health-statistics/overweight-obesity.

[27] Kamil-Rosenberg S, Kokkinos P, Grune de Souza E Silva C, Yee WLS, Abella J, Chan K (2020). Association between cardiorespiratory fitness, obesity, and incidence of atrial fibrillation. *International Journal of Cardiology. Heart & Vasculature.*.

[28] Garnvik LE, Malmo V, Janszky I, Wisløff U, Loennechen JP, Nes BM (2018). Physical activity modifies the risk of atrial fibrillation in obese individuals: The HUNT3 study. *European Journal of Preventive Cardiology*.

[29] Aker A, Saliba W, Zafrir B (2023). The interplay among body weight, blood pressure, and cardiorespiratory fitness in predicting atrial fibrillation. *Hellenic Journal of Cardiology*.

[30] Chao T, Liu C, Tuan T, Chen TJ, Hsieh MH, Lip GY (2018). Lifetime risks, projected numbers, and adverse outcomes in asian patients with atrial fibrillation: A report from the taiwan nationwide AF cohort study. *Chest*.

[31] Tamirisa KP, Al-Khatib SM, Mohanty S, Han JK, Natale A, Gupta D (2021). Racial and Ethnic Differences in the Management of Atrial Fibrillation. *CJC Open*.

[32] Alonso A, Agarwal SK, Soliman EZ, Ambrose M, Chamberlain AM, Prineas RJ (2009). Incidence of atrial fibrillation in whites and African-Americans: the Atherosclerosis Risk in Communities (ARIC) study. *American Heart Journal*.

[33] Volgman AS, Bairey Merz CN, Benjamin EJ, Curtis AB, Fang MC, Lindley KJ (2019). Sex and Race/Ethnicity Differences in Atrial Fibrillation. *Journal of the American College of Cardiology*.

[34] Heckbert SR, Austin TR, Jensen PN, Chen LY, Post WS, Floyd JS (2020). Differences by Race/Ethnicity in the Prevalence of Clinically Detected and Monitor-Detected Atrial Fibrillation: MESA. *Circulation. Arrhythmia and Electrophysiology.*.

[35] Simpson JR, Zahuranec DB, Lisabeth LD, Sánchez BN, Skolarus LE, Mendizabal JE (2010). Mexican Americans with atrial fibrillation have more recurrent strokes than do non-Hispanic whites. *Stroke*.

[36] Karjalainen J, Kujala UM, Kaprio J, Sarna S, Viitasalo M (1998). Lone atrial fibrillation in vigorously exercising middle aged men: case-control study. *BMJ (Clinical Research Ed.).*.

[37] Andersen K, Farahmand B, Ahlbom A, Held C, Ljunghall S, Michaëlsson K (2013). Risk of arrhythmias in 52 755 long-distance cross-country skiers: a cohort study. *European Heart Journal*.

[38] Svedberg N, Sundström J, James S, Hållmarker U, Hambraeus K, Andersen K (2019). Long-Term Incidence of Atrial Fibrillation and Stroke Among Cross-Country Skiers. *Circulation*.

[39] Baldesberger S, Bauersfeld U, Candinas R, Seifert B, Zuber M, Ritter M (2008). Sinus node disease and arrhythmias in the long-term follow-up of former professional cyclists. *European Heart Journal*.

[40] Myrstad M, Nystad W, Graff-Iversen S, Thelle DS, Stigum H, Aarønæs M (2014). Effect of years of endurance exercise on risk of atrial fibrillation and atrial flutter. *The American Journal of Cardiology*.

[41] Aagaard P, Sharma S, McNamara DA, Joshi P, Ayers CR, de Lemos JA (2019). Arrhythmias and Adaptations of the Cardiac Conduction System in Former National Football League Players. *Journal of the American Heart Association*.

[42] Boraita A, Santos-Lozano A, Heras ME, González-Amigo F, López-Ortiz S, Villacastín JP (2018). Incidence of Atrial Fibrillation in Elite Athletes. *JAMA Cardiology*.

[43] Shapira-Daniels A, Kornej J, Spartano NL, Wang X, Zhang Y, Pathiravasan CH (2023). Step Count, Self-reported Physical Activity, and Predicted 5-Year Risk of Atrial Fibrillation: Cross-sectional Analysis. *Journal of Medical Internet Research*.

[44] Myrstad M, Løchen ML, Graff-Iversen S, Gulsvik AK, Thelle DS, Stigum H (2014). Increased risk of atrial fibrillation among elderly Norwegian men with a history of long-term endurance sport practice. *Scandinavian Journal of Medicine & Science in Sports*.

[45] Johansen KR, Ranhoff AH, Sørensen E, Nes BM, Heitmann KA, Apelland T (2022). Risk of atrial fibrillation and stroke among older men exposed to prolonged endurance sport practice: a 10-year follow-up. *The Birkebeiner Ageing Study and the Tromsø Study. Open Heart.*.

[46] Khan H, Kunutsor S, Rauramaa R, Savonen K, Kalogeropoulos AP, Georgiopoulou VV (2014). Cardiorespiratory fitness and risk of heart failure: a population-based follow-up study. *European Journal of Heart Failure*.

[47] Mishima RS, Verdicchio CV, Noubiap JJ, Ariyaratnam JP, Gallagher C, Jones D (2021). Self-reported physical activity and atrial fibrillation risk: A systematic review and meta-analysis. *Heart Rhythm*.

[48] Kunutsor SK, Seidu S, Mäkikallio TH, Dey RS, Laukkanen JA (2021). Physical activity and risk of atrial fibrillation in the general population: meta-analysis of 23 cohort studies involving about 2 million participants. *European Journal of Epidemiology*.

[49] Newman W, Parry-Williams G, Wiles J, Edwards J, Hulbert S, Kipourou K (2021). Risk of atrial fibrillation in athletes: a systematic review and meta-analysis. *British Journal of Sports Medicine*.

[50] Elliott AD, Linz D, Mishima R, Kadhim K, Gallagher C, Middeldorp ME (2020). Association between physical activity and risk of incident arrhythmias in 402 406 individuals: evidence from the UK Biobank cohort. *European Heart Journal*.

[51] Tikkanen E, Gustafsson S, Ingelsson E (2018). Associations of Fitness, Physical Activity, Strength, and Genetic Risk With Cardiovascular Disease: Longitudinal Analyses in the UK Biobank Study. *Circulation*.

[52] Khurshid S, Weng LC, Al-Alusi MA, Halford JL, Haimovich JS, Benjamin EJ (2021). Accelerometer-derived physical activity and risk of atrial fibrillation. *European Heart Journal*.

[53] Khurshid S, Al-Alusi MA, Churchill TW, Guseh JS, Ellinor PT (2023). Accelerometer-Derived “Weekend Warrior” Physical Activity and Incident Cardiovascular Disease. *JAMA*.

[54] Jin MN, Yang PS, Song C, Yu HT, Kim TH, Uhm JS (2019). Physical Activity and Risk of Atrial Fibrillation: A Nationwide Cohort Study in General Population. *Scientific Reports*.

[55] Qureshi WT, Alirhayim Z, Blaha MJ, Juraschek SP, Keteyian SJ, Brawner CA (2015). Cardiorespiratory Fitness and Risk of Incident Atrial Fibrillation: Results From the Henry Ford Exercise Testing (FIT) Project. *Circulation*.

[56] Khan H, Kella D, Rauramaa R, Savonen K, Lloyd MS, Laukkanen JA (2015). Cardiorespiratory fitness and atrial fibrillation: A population-based follow-up study. *Heart Rhythm*.

[57] Morseth B, Graff-Iversen S, Jacobsen BK, Jørgensen L, Nyrnes A, Thelle DS (2016). Physical activity, resting heart rate, and atrial fibrillation: the Tromsø Study. *European Heart Journal*.

[58] Mozaffarian D, Furberg CD, Psaty BM, Siscovick D (2008). Physical activity and incidence of atrial fibrillation in older adults: the cardiovascular health study. *Circulation*.

[59] Drca N, Wolk A, Jensen-Urstad M, Larsson SC (2014). Atrial fibrillation is associated with different levels of physical activity levels at different ages in men. *Heart*.

[60] Drca N, Wolk A, Jensen-Urstad M, Larsson SC (2015). Physical activity is associated with a reduced risk of atrial fibrillation in middle-aged and elderly women. *Heart*.

[61] Lee PH, Macfarlane DJ, Lam TH, Stewart SM (2011). Validity of the international physical activity questionnaire short form (IPAQ-SF): A systematic review. *International Journal of Behavioral Nutrition and Physical Activity*.

[62] Wan Q, Zhou Y, Zhu W, Liu X (2021). Sex-Specific Exposure-Effect Relationship Between Physical Activity and Incident Atrial Fibrillation in the General Population: A Dose-Response Meta-Analysis of 16 Prospective Studies. *Frontiers in Cardiovascular Medicine*.

[63] Magnani JW, Wang N, Benjamin EJ, Garcia ME, Bauer DC, Butler J (2016). Atrial Fibrillation and Declining Physical Performance in Older Adults: The Health, Aging, and Body Composition Study. *Circulation. Arrhythmia and Electrophysiology*.

[64] De Bosscher R, Dausin C, Claus P, Bogaert J, Dymarkowski S, Goetschalckx K (2021). Endurance exercise and the risk of cardiovascular pathology in men: a comparison between lifelong and late-onset endurance training and a non-athletic lifestyle - rationale and design of the Master@Heart study, a prospective cohort trial. *BMJ Open Sport & Exercise Medicine*.

[65] De Bosscher R, Dausin C, Janssens K, Bogaert J, Elliott A, Ghekiere O (2022). Rationale and design of the PROspective ATHletic Heart (Pro@Heart) study: long-term assessment of the determinants of cardiac remodelling and its clinical consequences in endurance athletes. *BMJ Open Sport & Exercise Medicine*.

[66] Apelland T, Janssens K, Loennechen JP, Claessen G, Sørensen E, Mitchell A (2023). Effects of training adaption in endurance athletes with atrial fibrillation: protocol for a multicentre randomised controlled trial. *BMJ Open Sport & Exercise Medicine*.

[67] Colombo CSSS, Finocchiaro G (2018). The Female Athlete’s Heart: Facts and Fallacies. *Current Treatment Options in Cardiovascular Medicine*.

[68] Wiebe CG, Gledhill N, Warburton DE, Jamnik VK, Ferguson S (1998). Exercise cardiac function in endurance-trained males versus females. *Clinical Journal of Sport Medicine*.

[69] Mont L, Tamborero D, Elosua R, Molina I, Coll-Vinent B, Sitges M (2008). Physical activity, height, and left atrial size are independent risk factors for lone atrial fibrillation in middle-aged healthy individuals. *Europace*.

[70] Rao SJ, Shah AB (2022). Exercise and the Female Heart. *Clinical Therapeutics*.

[71] Tannenbaum C, Norris CM, McMurtry MS (2019). Sex-Specific Considerations in Guidelines Generation and Application. *The Canadian Journal of Cardiology*.

[72] Garnvik LE, Malmo V, Janszky I, Wisløff U, Loennechen JP, Nes BM (2019). Estimated Cardiorespiratory Fitness and Risk of Atrial Fibrillation: The Nord-Trøndelag Health Study. *Medicine and Science in Sports and Exercise*.

[73] Azarbal F, Stefanick ML, Salmoirago-Blotcher E, Manson JE, Albert CM, LaMonte MJ (2014). Obesity, physical activity, and their interaction in incident atrial fibrillation in postmenopausal women. *Journal of the American Heart Association*.

[74] Fletcher G, Alam AB, Li L, Norby FL, Chen LY, Soliman EZ (2022). Association of physical activity with the incidence of atrial fibrillation in persons > 65 years old: the Atherosclerosis Risk in Communities (ARIC) study. *BMC Cardiovascular Disorders*.

[75] Mohanty S, Mohanty P, Tamaki M, Natale V, Gianni C, Trivedi C (2016). Differential Association of Exercise Intensity With Risk of Atrial Fibrillation in Men and Women: Evidence from a Meta-Analysis. *Journal of Cardiovascular Electrophysiology*.

[76] Anagnostopoulos I, Kousta M, Kossyvakis C, Lakka E, Vrachatis D, Deftereos S (2023). Weekly physical activity and incident atrial fibrillation in females - A dose-response meta-analysis. *International Journal of Cardiology*.

[77] Kavousi M (2020). Differences in Epidemiology and Risk Factors for Atrial Fibrillation Between Women and Men. *Frontiers in Cardiovascular Medicine*.

[78] Wang R, Olier I, Ortega-Martorell S, Liu Y, Ye Z, Lip GY (2022). Association between metabolically healthy obesity and risk of atrial fibrillation: taking physical activity into consideration. *Cardiovascular Diabetology*.

[79] Sharashova E, Gerdts E, Ball J, Espnes H, Jacobsen BK, Kildal S (2023). Sex-specific time trends in incident atrial fibrillation and the contribution of risk factors: the Tromsø Study 1994-2016. *European Journal of Preventive Cardiology*.

[80] Myrstad M, Sørensen E, Janssens K, Mitchell A, Apelland T, Claessen G (2023). Exercise-Induced Cardiac Remodeling and Atrial Fibrillation in Female Endurance Athletes. *Journal of the American Society of Echocardiography*.

[81] Myrstad M, Aarønæs M, Graff-Iversen S, Nystad W, Ranhoff AH (2015). Does endurance exercise cause atrial fibrillation in women?. *International Journal of Cardiology*.

[82] Myrstad M, Johansen KR, Sørensen E, Løchen ML, Ranhoff AH, Morseth B (2024). Atrial fibrillation in female endurance athletes. *European Journal of Preventive Cardiology*.

[83] Drca N, Larsson SC, Grannas D, Jensen-Urstad M (2023). Elite female endurance athletes are at increased risk of atrial fibrillation compared to the general population: a matched cohort study. *British Journal of Sports Medicine*.

[84] Shih T, Ledezma K, McCauley M, Rehman J, Galanter WL, Darbar D (2020). Impact of traditional risk factors for the outcomes of atrial fibrillation across race and ethnicity and sex groups. *International Journal of Cardiology. Heart & Vasculature.*.

[85] Mou L, Norby FL, Chen LY, O’Neal WT, Lewis TT, Loehr LR (2018). Lifetime Risk of Atrial Fibrillation by Race and Socioeconomic Status: ARIC Study (Atherosclerosis Risk in Communities). *Circulation. Arrhythmia and Electrophysiology*.

[86] Rodriguez CJ, Soliman EZ, Alonso A, Swett K, Okin PM, Goff DC (2015). Atrial fibrillation incidence and risk factors in relation to race-ethnicity and the population attributable fraction of atrial fibrillation risk factors: the Multi-Ethnic Study of Atherosclerosis. *Annals of Epidemiology*.

[87] Huang H, Darbar D (2017). Genetic heterogeneity of atrial fibrillation susceptibility loci across racial or ethnic groups. *European Heart Journal*.

[88] Schnabel RB, Yin X, Gona P, Larson MG, Beiser AS, McManus DD (2015). 50 year trends in atrial fibrillation prevalence, incidence, risk factors, and mortality in the Framingham Heart Study: a cohort study. *Lancet*.

[89] Heeringa J, van der Kuip DAM, Hofman A, Kors JA, van Herpen G, Stricker BHC (2006). Prevalence, incidence and lifetime risk of atrial fibrillation: the Rotterdam study. *European Heart Journal*.

[90] O’Neal WT, Bennett A, Singleton MJ, Judd SE, Howard G, Howard VJ (2020). Objectively Measured Physical Activity and the Risk of Atrial Fibrillation (from the REGARDS Study). *The American Journal of Cardiology*.

[91] Hunter GR, Weinsier RL, Darnell BE, Zuckerman PA, Goran MI (2000). Racial differences in energy expenditure and aerobic fitness in premenopausal women. *The American Journal of Clinical Nutrition*.

[92] Hunter GR, Weinsier RL, McCarthy JP, Enette Larson-Meyer D, Newcomer BR (2001). Hemoglobin, muscle oxidative capacity, and VO2max in African-American and Caucasian women. *Medicine and Science in Sports and Exercise*.

[93] Toledo FGS, Dubé JJ, Goodpaster BH, Stefanovic-Racic M, Coen PM, DeLany JP (2018). Mitochondrial Respiration is Associated with Lower Energy Expenditure and Lower Aerobic Capacity in African American Women. *Obesity*.

[94] McEligot AJ, Mitra S, Beam W (2021). The association between fitness and obesity in diverse multi-ethnic college students. *Journal of American College Health*.

[95] Sabbahi A, Arena R, Kaminsky LA, Myers J, Fernhall B, Sundeep C (2021). Characterization of the blood pressure response during cycle ergometer cardiopulmonary exercise testing in black and white men: Data from the Fitness Registry and Importance of Exercise: A National Database (FRIEND). *Journal of Human Hypertension*.

[96] Bazargan-Hejazi S, Arroyo JS, Hsia S, Brojeni NR, Pan D (2017). A Racial Comparison of Differences between Self-Reported and Objectively Measured Physical Activity among US Adults with Diabetes. *Ethnicity & Disease*.

[97] Visaria A, Nagaraj B, Shah M, Kethidi N, Modak A, Shahani J (2022). Low Amount and Intensity of Leisure-time Physical Activity in Asian Indian Adults. *American Journal of Health Promotion*.

[98] Saffer H, Dave D, Grossman M, Leung LA (2013). Racial, Ethnic, and Gender Differences in Physical Activity. *Journal of Human Capital*.

[99] Al-Mallah MH, Qureshi WT, Keteyian SJ, Brawner CA, Alam M, Dardari Z (2016). Racial Differences in the Prognostic Value of Cardiorespiratory Fitness (Results from the Henry Ford Exercise Testing Project). *The American Journal of Cardiology*.

[100] Vásquez E, Sahakyan K, Batsis JA, Germain C, Somers VK, Shaw BA (2018). Ethnic differences in all-cause and cardiovascular mortality by physical activity levels among older adults in the US. *Ethnicity & Health*.

[101] Carnethon MR, Pu J, Howard G, Albert MA, Anderson CAM, Bertoni AG (2017). Cardiovascular Health in African Americans: A Scientific Statement From the American Heart Association. *Circulation*.

[102] Boyer WR, Bassett DR, Fitzhugh EC, Milano AN, Churilla JR, Toth LP (2022). Accelerometer-Measured Physical Activity and Cardiometabolic Risk Factors by Race-Ethnicity: 2003-2006 NHANES. *Journal of Racial and Ethnic Health Disparities*.

[103] Brewer LC, Jenkins S, Hayes SN, Kumbamu A, Jones C, Burke LE (2022). Community-Based, Cluster-Randomized Pilot Trial of a Cardiovascular Mobile Health Intervention: Preliminary Findings of the FAITH! Trial. *Circulation*.

[104] Joseph RP, Todd M, Ainsworth BE, Vega-López S, Adams MA, Hollingshead K (2023). *Smart Walk*: A Culturally Tailored Smartphone-Delivered Physical Activity Intervention for Cardiometabolic Risk Reduction among African American Women. *International Journal of Environmental Research and Public Health*.

[105] Wilbur J, Miller AM, Fogg L, McDevitt J, Castro CM, Schoeny ME (2016). Randomized Clinical Trial of the Women’s Lifestyle Physical Activity Program for African-American Women: 24- and 48-Week Outcomes. *American Journal of Health Promotion*.

[106] Schulz AJ, Israel BA, Mentz GB, Bernal C, Caver D, DeMajo R (2015). Effectiveness of a walking group intervention to promote physical activity and cardiovascular health in predominantly non-Hispanic black and Hispanic urban neighborhoods: findings from the walk your heart to health intervention. *Health Education & Behavior*.

[107] Chee W, Kim S, Tsai HM, Liu J, Im EO (2020). Effect of An Online Physical Activity Promotion Program and Cardiovascular Symptoms Among Asian American Women at Midlife. *Computers, Informatics, Nursing*.

[108] Kaholokula JK, Look M, Mabellos T, Ahn HJ, Choi SY, Sinclair KA (2021). A Cultural Dance Program Improves Hypertension Control and Cardiovascular Disease Risk in Native Hawaiians: A Randomized Controlled Trial. *Annals of Behavioral Medicine*.

[109] Kandula NR, Dave S, De Chavez PJ, Bharucha H, Patel Y, Seguil P (2015). Translating a heart disease lifestyle intervention into the community: the South Asian Heart Lifestyle Intervention (SAHELI) study; a randomized control trial. *BMC Public Health*.

[110] Rosas LG, Lv N, Azar KMJ, Xiao L, Hooker SP, Lewis MA (2018). HOMBRE: A randomized controlled trial to compare two approaches to weight loss for overweight and obese Latino men (Hombres con Opciones para Mejorar el Bienestar y bajar el Riesgo de Enfermedades crónicas; men with choices to improve well-being and decrease chronic disease risk). *Contemporary Clinical Trials*.

[111] Guasch E, Mont L, Sitges M (2018). Mechanisms of atrial fibrillation in athletes: what we know and what we do not know. *Netherlands Heart Journal*.

[112] Stergiou D, Duncan E (2018). Atrial Fibrillation (AF) in Endurance Athletes: a Complicated Affair. *Current Treatment Options in Cardiovascular Medicine*.

[113] Heitmann KA, Løchen ML, Stylidis M, Hopstock LA, Schirmer H, Morseth B (2022). Associations between physical activity, left atrial size and incident atrial fibrillation: the Tromsø Study 1994-2016. *Open Heart*.

[114] D’Ascenzi F, Cameli M, Ciccone MM, Maiello M, Modesti PA, Mondillo S (2015). The controversial relationship between exercise and atrial fibrillation: clinical studies and pathophysiological mechanisms. *Journal of Cardiovascular Medicine*.

[115] Utomi V, Oxborough D, Whyte GP, Somauroo J, Sharma S, Shave R (2013). Systematic review and meta-analysis of training mode, imaging modality and body size influences on the morphology and function of the male athlete’s heart. *Heart*.

[116] Trivedi SJ, Claessen G, Stefani L, Flannery MD, Brown P, Janssens K (2020). Differing mechanisms of atrial fibrillation in athletes and non-athletes: alterations in atrial structure and function. *European Heart Journal. Cardiovascular Imaging*.

[117] Sørensen E, Myrstad M, Solberg MG, Øie E, Tveit A, Aarønæs M (2021). Left atrial function in male veteran endurance athletes with paroxysmal atrial fibrillation. *European Heart Journal. Cardiovascular Imaging.*.

[118] Sørensen E, Myrstad M, Solberg MG, Øie E, Platonov PG, Carlson J (2023). Left atrial dyssynchrony in veteran endurance athletes with and without paroxysmal atrial fibrillation. *Echocardiography*.

[119] Sørensen E, Myrstad M, Solberg MG, Øie E, Tveit A, Aarønæs M (2022). Right Heart Structure and Function in Lifelong Recreational Endurance Athletes with and without Paroxysmal Atrial Fibrillation. *Journal of the American Society of Echocardiography*.

[120] Elliott AD, Middeldorp ME, Van Gelder IC, Albert CM, Sanders P (2023). Epidemiology and modifiable risk factors for atrial fibrillation. *Nature Reviews. Cardiology.*.

[121] Fulghum K, Hill BG (2018). Metabolic Mechanisms of Exercise-Induced Cardiac Remodeling. *Frontiers in Cardiovascular Medicine*.

[122] Wilhelm M, Roten L, Tanner H, Wilhelm I, Schmid JP, Saner H (2011). Gender differences of atrial and ventricular remodeling and autonomic tone in nonelite athletes. *The American Journal of Cardiology*.

[123] Dempsey PC, Larsen RN, Dunstan DW, Owen N, Kingwell BA (2018). Sitting Less and Moving More: Implications for Hypertension. *Hypertension*.

[124] Ihara K, Sasano T (2022). Role of Inflammation in the Pathogenesis of Atrial Fibrillation. *Frontiers in Physiology*.

[125] Packer M (2018). Epicardial Adipose Tissue May Mediate Deleterious Effects of Obesity and Inflammation on the Myocardium. *Journal of the American College of Cardiology*.

[126] Abe I, Teshima Y, Kondo H, Kaku H, Kira S, Ikebe Y (2018). Association of fibrotic remodeling and cytokines/chemokines content in epicardial adipose tissue with atrial myocardial fibrosis in patients with atrial fibrillation. *Heart Rhythm*.

[127] Christensen RH, Wedell-Neergaard AS, Lehrskov LL, Legaard GE, Dorph E, Larsen MK (2019). Effect of Aerobic and Resistance Exercise on Cardiac Adipose Tissues: Secondary Analyses From a Randomized Clinical Trial. *JAMA Cardiology*.

[128] Aune D, Norat T, Leitzmann M, Tonstad S, Vatten LJ (2015). Physical activity and the risk of type 2 diabetes: a systematic review and dose-response meta-analysis. *European Journal of Epidemiology*.

[129] Boulé NG, Haddad E, Kenny GP, Wells GA, Sigal RJ (2001). Effects of exercise on glycemic control and body mass in type 2 diabetes mellitus: a meta-analysis of controlled clinical trials. *JAMA*.

[130] Mayer-Davis EJ, D’Agostino R, Karter AJ, Haffner SM, Rewers MJ, Saad M (1998). Intensity and amount of physical activity in relation to insulin sensitivity: the Insulin Resistance Atherosclerosis Study. *JAMA*.

[131] Kwon S, Lee SR, Choi EK, Lee SW, Jung JH, Han KD (2024). Impact of Unhealthy Lifestyles on Patients with Atrial Fibrillation at Low Risk of Stroke: A Nationwide Cohort Study. *The American Journal of Medicine*.

[132] Cauwenberghs N, Sente J, Sabovčik F, Ntalianis E, Hedman K, Claes J (2023). Cardiorespiratory fitness components in relation to clinical characteristics, disease state and medication intake: A patient registry study. *Clinical Physiology and Functional Imaging*.

[133] Buckley BJR, Harrison SL, Fazio-Eynullayeva E, Underhill P, Lane DA, Thijssen DHJ (2021). Exercise-Based Cardiac Rehabilitation and All-Cause Mortality Among Patients With Atrial Fibrillation. *Journal of the American Heart Association*.

[134] Donnellan E, Wazni OM, Harb S, Kanj M, Saliba WI, Jaber WA (2020). Higher baseline cardiorespiratory fitness is associated with lower arrhythmia recurrence and death after atrial fibrillation ablation. *Heart Rhythm*.

[135] Christensen SW, Berg SK, Rod NH, Zwisler ADO, Thygesen LC, Risom SS (2021). Physical activity and serious adverse events in patients with atrial fibrillation and/or atrial flutter treated with catheter ablation. *Heart & Lung*.

[136] Semaan S, Dewland TA, Tison GH, Nah G, Vittinghoff E, Pletcher MJ (2020). Physical activity and atrial fibrillation: Data from wearable fitness trackers. *Heart Rhythm*.

[137] Ahn HJ, Lee SR, Choi EK, Han KD, Jung JH, Lim JH (2021). Association between exercise habits and stroke, heart failure, and mortality in Korean patients with incident atrial fibrillation: A nationwide population-based cohort study. *PLoS Medicine*.

[138] Hegbom F, Stavem K, Sire S, Heldal M, Orning OM, Gjesdal K (2007). Effects of short-term exercise training on symptoms and quality of life in patients with chronic atrial fibrillation. *International Journal of Cardiology*.

[139] Osbak PS, Mourier M, Kjaer A, Henriksen JH, Kofoed KF, Jensen GB (2011). A randomized study of the effects of exercise training on patients with atrial fibrillation. *American Heart Journal*.

[140] Risom SS, Zwisler AD, Rasmussen TB, Sibilitz KL, Madsen TLS, Svendsen JH (2016). Cardiac rehabilitation versus usual care for patients treated with catheter ablation for atrial fibrillation: Results of the randomized CopenHeart_RFA_ trial. *American Heart Journal*.

[141] Malmo V, Nes BM, Amundsen BH, Tjonna AE, Stoylen A, Rossvoll O (2016). Aerobic Interval Training Reduces the Burden of Atrial Fibrillation in the Short Term: A Randomized Trial. *Circulation*.

[142] Kato M, Ogano M, Mori Y, Kochi K, Morimoto D, Kito K (2019). Exercise-based cardiac rehabilitation for patients with catheter ablation for persistent atrial fibrillation: A randomized controlled clinical trial. *European Journal of Preventive Cardiology*.

[143] Elliott AD, Verdicchio CV, Mahajan R, Middeldorp ME, Gallagher C, Mishima RS (2023). An Exercise and Physical Activity Program in Patients With Atrial Fibrillation: The ACTIVE-AF Randomized Controlled Trial. *JACC. Clinical Electrophysiology.*.

[144] Alves LS, Bocchi EA, Chizzola PR, Castro RE, Salemi VMC, de Melo MDT (2022). Exercise training in heart failure with reduced ejection fraction and permanent atrial fibrillation: A randomized clinical trial. *Heart Rhythm*.

[145] Oesterle A, Giancaterino S, Van Noord MG, Pellegrini CN, Fan D, Srivatsa UN (2022). Effects of Supervised Exercise Training on Atrial Fibrillation: A META-ANALYSIS OF RANDOMIZED CONTROLLED TRIALS. *Journal of Cardiopulmonary Rehabilitation and Prevention*.

[146] Skielboe AK, Bandholm TQ, Hakmann S, Mourier M, Kallemose T, Dixen U (2017). Cardiovascular exercise and burden of arrhythmia in patients with atrial fibrillation - A randomized controlled trial. *PLoS ONE*.

[147] Pathak RK, Elliott A, Middeldorp ME, Meredith M, Mehta AB, Mahajan R (2015). Impact of CARDIOrespiratory FITness on Arrhythmia Recurrence in Obese Individuals With Atrial Fibrillation: The CARDIO-FIT Study. *Journal of the American College of Cardiology*.

[148] Pathak RK, Middeldorp ME, Meredith M, Mehta AB, Mahajan R, Wong CX (2015). Long-Term Effect of Goal-Directed Weight Management in an Atrial Fibrillation Cohort: A Long-Term Follow-Up Study (LEGACY). *Journal of the American College of Cardiology*.

[149] Rienstra M, Hobbelt AH, Alings M, Tijssen JGP, Smit MD, Brügemann J (2018). Targeted therapy of underlying conditions improves sinus rhythm maintenance in patients with persistent atrial fibrillation: results of the RACE 3 trial. *European Heart Journal*.

[150] Reed JL, Terada T, Vidal-Almela S, Tulloch HE, Mistura M, Birnie DH (2022). Effect of High-Intensity Interval Training in Patients With Atrial Fibrillation: A Randomized Clinical Trial. *JAMA Network Open*.

[151] Myrstad M, Malmo V, Ulimoen SR, Tveit A, Loennechen JP (2019). Exercise in individuals with atrial fibrillation. *Clinical Research in Cardiology*.

